# Comparison of human Mesenchymal Stem Cells biocompatibility data growth on gelatin and silk fibroin scaffolds

**DOI:** 10.1016/j.dib.2019.104678

**Published:** 2019-10-17

**Authors:** Noviana Vanawati, Anggraini Barlian, Yasuhiko Tabata, Hermawan Judawisastra, Indra Wibowo

**Affiliations:** a School of Life Science and Technology, Institut Teknologi Bandung, Bandung, Indonesia; b Laboratory of Biomaterials, Department of Regeneration Science and Engineering, Institute for Frontier Life and Medical Sciences, Kyoto University, Kyoto, Japan; c Materials Science and Engineering Department, Faculty of Mechanical and Aerospace Engineering, Institut Teknologi Bandung, Bandung, Indonesia

**Keywords:** Gelatin hydrogel, Silk fibroin scaffold, Human mesenchymal stem cells, Biocompatibility

## Abstract

The data showed how gelatin hydrogel and silk fibroin scaffolds could facilitate the growth of human Mesenchymal Stem Cells (hMSC). Gelatin hydrogel and silk fibroin are biodegradable materials. Gelatin hydrogel already has many uses in the medical field, especially in tissue engineering, but silk fibroin scaffold, which is made from the cocoon of silkworm by salt leaching, its role in facilitating growth of hMSC still needs to be proven. Data was obtained by characterization of hMSC, then growing hMSC on silk fibroin scaffolds with pore sizes of ±500 μm and ±900 μm and on gelatin hydrogel scaffolds as control. Testing was performed by counting cell growth on days 1, 3, 5, 7 and 14 with the MTT cytotoxicity assay protocol. The morphology of hMSC that grew on gelatin and silk fibroin scaffolds was observed with a Scanning Electron Microscope (SEM) on day 3. Characterization of the hMSC showed that it fulfilled the requirements of the International Society for Cellular Therapy (ISCT). The water content of the gelatin hydrogel scaffold was higher than the silk fibroin scaffold. Biocompatibility testing showed that the gelatin hydrogel scaffold could support cell growth until day 7, then decreased on day 14 compared to the silk fibroin scaffold based on absorbance on the MTT cytotoxicity assay, while growth on silk fibroin scaffold with pore size 833 ± 147 μm was consistently higher than on pore size 462 ± 66 μm from day 1 to day 14. Cell binding to the silk fibroin was proven from SEM observation.

**Table:** Specifications Table

Subject	Biomaterials
Specific subject area	Gelatin hydrogel scaffold and silk fibroin scaffold
Type of data	Image Chart
How data were acquired	hMSC characterization (Phase contrast microscope and BD Accuri™ C6 flow cytometer) MTT cytotoxic assay (Bio-rad microplate reader) Scaffold and cell morphology (Scanning Electron Microscope, SU3500 Hitachi High Technologies America, Inc).
Data format	Raw data analyzed
Parameters for data collection	hMSC that were used fulfilled criteria according to ISCT standard and could proliferate when inoculated onto gelatin hydrogel scaffold and silk fibroin scaffold.
Description of data collection	Characterization of hMSC was performed by cell expansion on polystyrene substrate, then characterized for cell adhesion, antigen surface analysis, and multidifferentiation test. hMSC were then inoculated onto the scaffolds and incubated for 14 days. Biocompatibility tests were performed by MTT cytotoxicity assay on days 1, 3, 5, 7, and 14, and observation of morphology with SEM on day 3.
Data source location	Institut Teknologi Bandung Bandung, West Java Indonesia Latitude −6° 53′ 23.47″ S; Longitude 107° 36′ 35.88″ E
Data accessibility	Within this article

**Table:** 

**Value of the Data** • This data is useful to find a scaffold that is compatible for further applications in tissue engineering. • This data is useful for researchers working in the field of biomaterial and tissue engineering. • From this research, it is hoped that the use of gelatin hydrogel and silk fibroin biomaterials as scaffolds would not only facilitate cell growth, but would also support hMSC differentiation. • The novelty of the data was in the salt leaching method to make small (500 μm) and large (900 μm) pores in the silk fibroin scaffold, which then would be used to observe which pore size would be more suitable to facilitate cell growth.

## 1 Data

**Fig. 1:**
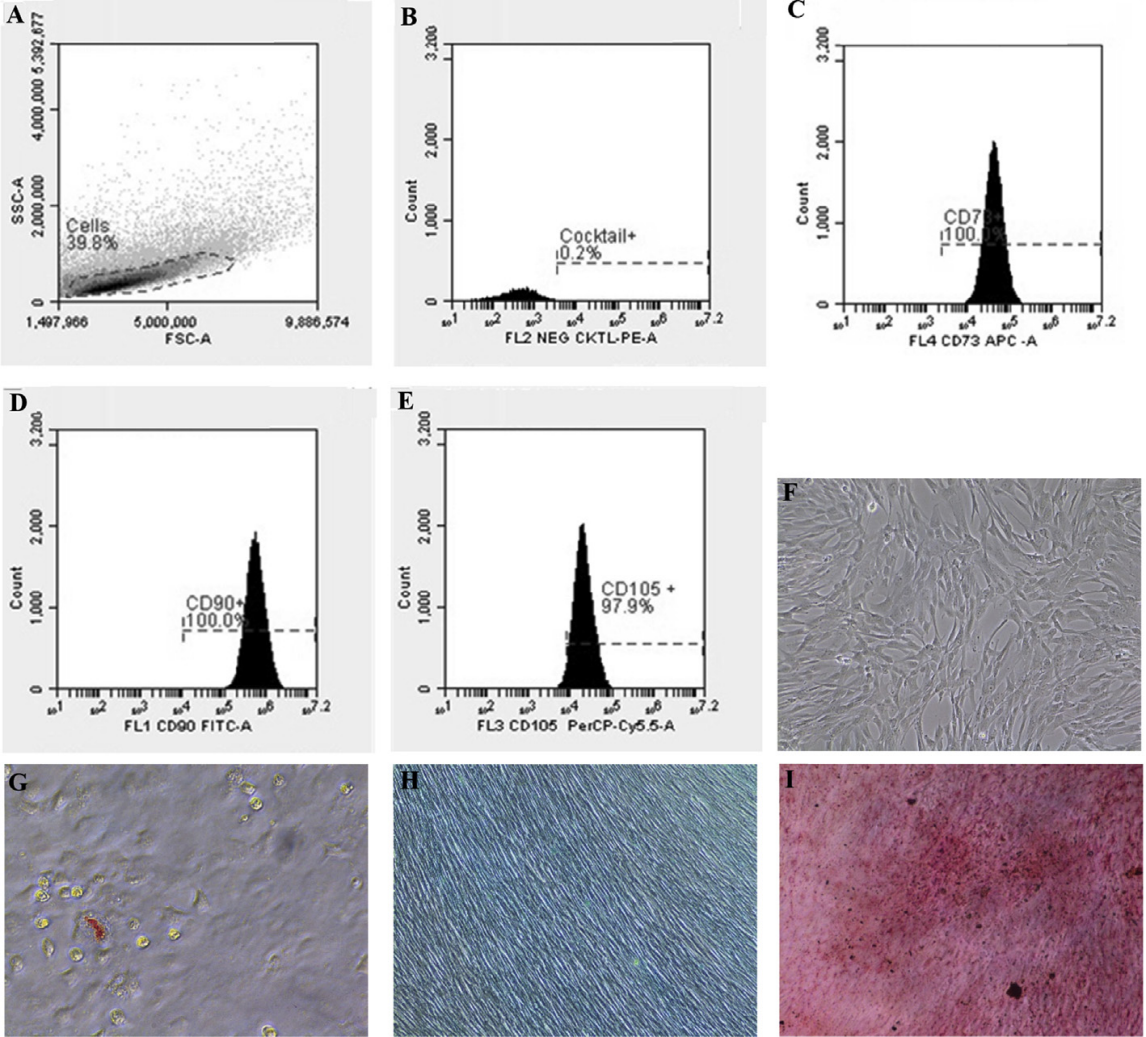
The specific cell surface markers analysis within the plotting cells (A); negative markers: CD45, CD34, CD11b, CD19, HLA-DR (B); positive markers: CD73 (C), CD 90(D), CD 105 (E). The hMSC morphology in complete culture medium (F). Multipotency Evaluation with Oil Red O Staining (G), Alcian Blue Staining (H) and Alizarin Red Staining (I) of hMSC.

**Fig. 2:**
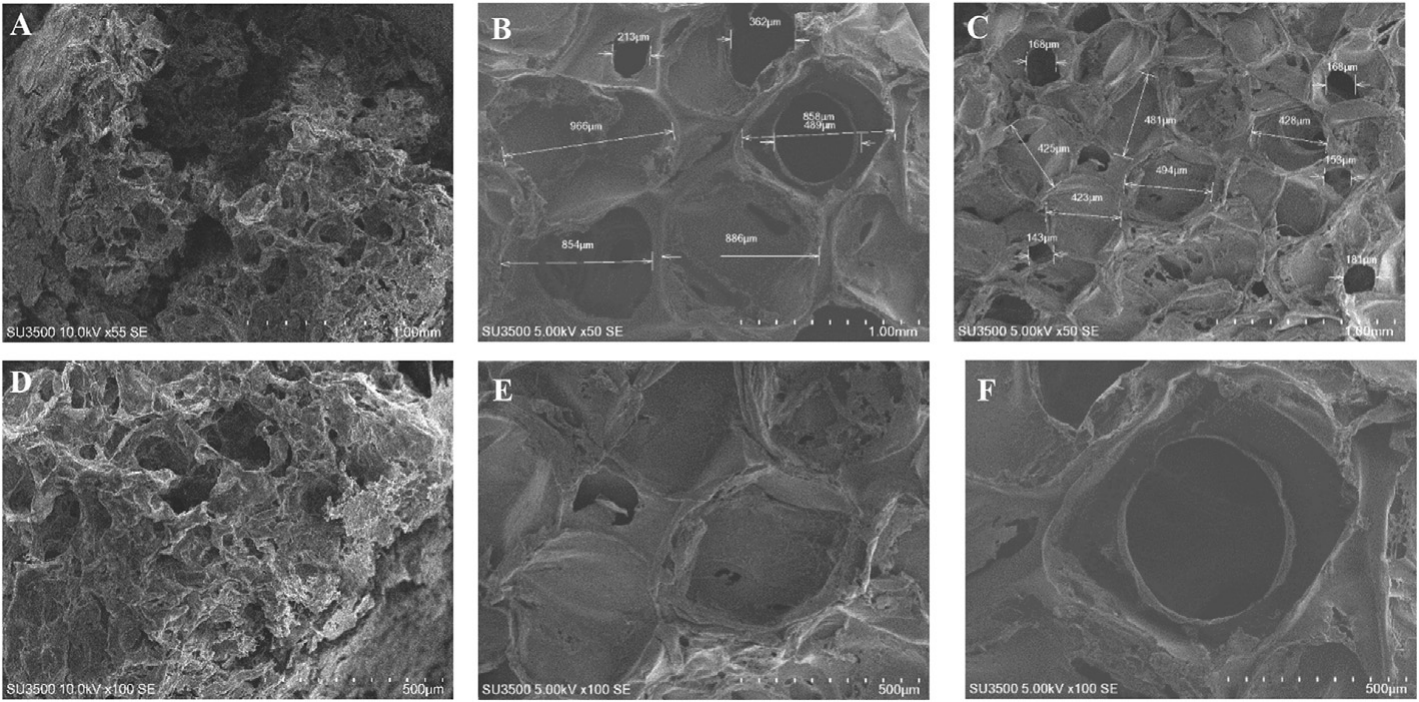
Morphology of gelatin scaffold (A,D); 12% w/v silk fibroin scaffold; pore size 462 ± 66 μm (B, E), pore size 833 ± 147 μm (D, F) observed with SEM.

**Fig. 3:**
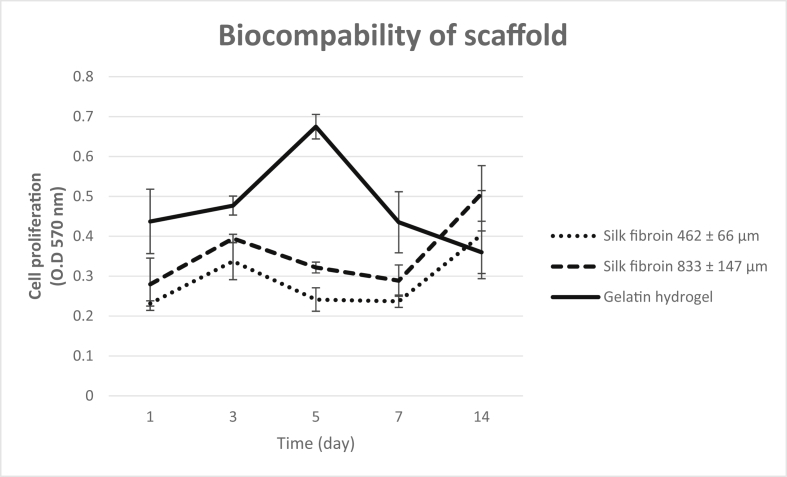
Growth curve of hMSC on various scaffolds (gelatin hydrogel, silk fibroin pore size 462 ± 66 μm on silk fibroin pore size 833 ± 147 μm) on days 1, 3, 5, 7, and 14 in standard culture condition.

**Fig. 4:**
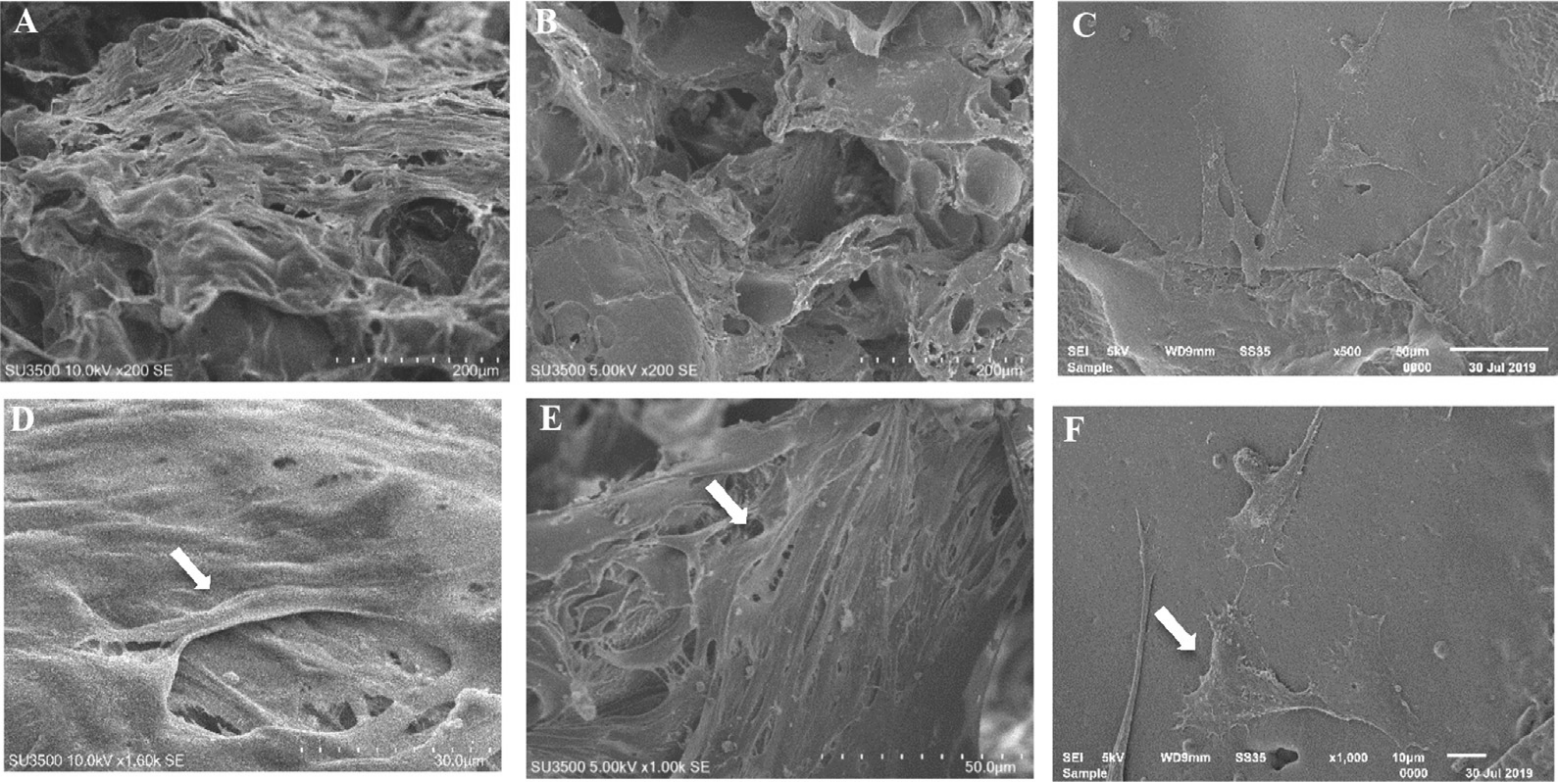
SEM images of hMSC morphology on gelatin hydrogel (A, D), silk fibroin pore size 462 ± 66 μm (B, E) and silk fibroin pore size 833 ± 147 μm (C, F) on day 3 in standard culture condition observed with SEM. Cells is shown by white arrows.

First data, we present the characterization data of human Mesenchymal Stem Cells (hMSC) based on the requirements of the International Society for Cellular Therapy (ISCT) in Fig. 1 hMSC had a negative specific cell surface markers <2% (Fig. 1B), positive specific cell surface maker >90% (Fig. 1C, D, E) using flow cytometry method. hMSC also had a flattened fibroblastic morphology and able to attached to the substrate in complete culture medium (Fig. 1F) and able to differentiate into three different types of cells, adypoctyes with Oil Red O Staining (Fig. 1G), Chondrocytes with Alcian Blue Staining (Fig. 1H) and Osteocyte with Alizarin Red Staining (Fig. 1I). The morphology and pores of scaffold using Scanning Electron Microscope (SEM) are shown in Fig. 2 and water content of scaffolds shown in Table 1. Gelatin and silk fibroin scaffold was not toxic for the hMSC and can support the cells growth on 1,3,5,7,14 days (Fig. 3) and cell morphology was observed with SEM (Fig. 4).

**Table 1:** Water content of scaffold (%).

Observation time (hours)	Scaffold		
	Gelatin Hydrogel	Silk Fibroin 462 ± 66 μm	Silk Fibroin 833 ± 147 μm
1	97.454	79.865	87.527
	97.426	79.897	88.621
	97.140	81.053	87.984
24	97.476	84.770	89.180
	97.865	82.541	88.788
	97.140	81.129	92.585

## 2 Experimental design, materials, and methods

### 2.1 Preparation of hMSC culture

#### 2.1.1 Isolation of human Mesenchymal Stem Cells (hMSC)

Human Mesenchymal Stem Cells (hMSC) were obtained from the umbilical cord of newborns and had already passed the ethical clearance from the Faculty of Medicine, Universitas Gadjah Mada. Cells were isolated with the explant method in standard culture medium composed of DMEM low glucose medium (Gibco 11885092), 10% Fetal Bovine Serum (Gibco 10270098), 1% Antibiotic-antimycotic (Gibco 15240062). The culture was incubated in an incubator at 37 °C and 5% CO_2_. When the hMSC became 70–80% confluent, the cells were subcultured for the cell expansion process by enzymatic treatment with Trypsin-EDTA (0,05%) (Gibco 25300054). *Cell harvesting* was performed on passage 5 hMSC for the research testing.

### 2.2 Characterization of hMSC in accordance with the International Society for Cellular Therapy (ISCT)

#### 2.2.1 Analysis of surface specific antigen expression on hMSC

hMSC passage 5 (1 × 10^6^ cells) were suspended in 1 mL phosphate buffered saline (PBS) sterile solution, pH 7.4, then 100 μL hMSC in PBS were transferred into five different analysis tubes in accordance with the protocol of the Human MSC Analysis Kit, BD Stemflow™. The tubes were incubated in the dark for 30 minutes at room temperature, then they were washed twice with PBS. The cells were resuspended in 500 μL PBS and analyzed with BD Accuri™ C6 Flowcytometer. The cells had to fulfill the criteria of ISCT: the mesenchymal stem cells had to have Cell Surface Markers (>95%) CD105, CD 90 and CD 73, and should not express (<2%) CD 45, CD 34, CD 14 or CD IB, CD 74 or CD 19, HLA class II.

#### 2.2.2 Analysis of multipotential differentiation

hMSC passage 5 were cultured in 24 well plate at 1 × 10^4^ cells/well in standard medium. After 70%–80% confluence, the standard medium was changed with adipogenic differentiation (Stempro™ Adipogenesis Differentiation kit, Gibco A1007001), chondrogenic (Stempro™ Chondrogenesis Differentiation kit, Gibco A1007101), and osteogenic (Stempro™ Osteogenesis Differentiation kit, Gibco A1007201) induction media. After 21 days, the process of cell staining was performed by fixation using 4% formaldehyde followed by treatment with Oil Red O for adipocyte specific staining, Alcian Blue for chondrocyte specific staining and Alizarin Red for osteocyte specific staining. Differentiation of hMSC into adipocyte, chondrocyte and osteocyte was observed under the microscope.

### 2.3 Biomaterial fabrication

#### 2.3.1 Biomaterial from silk fibroin

The silk fibroin scaffold was made using the salt leaching method as described by Wibowo et al. (2019) and Barlian et al. (2019) [1,2]. Silkworm (*Bombyx mori*) cocoons were obtained from CV. Wisata Ilmu Sutera, Bandung. Degumming to remove sericin from the cocoon was performed by soaking the cocoons in 0.05% NaHCO_3_ (PT. Bratachem, Bandung) for one hour, followed by washing in deionized water. The degumming process was repeated twice. The isolated silk fibroin was dried in a fume hood overnight. A 12 w/v% silk fibroin solution was made by dissolving the dried silk fibroin in 8 wt% CaCl_2_-formic acid (PT. Bratachem, Bandung) solution at room temperature using a magnetic stirrer for 15–30 minutes. The pores were formed by adding NaCl (Sakura Medical Dental Laboratorium & Chemical, Bandung) with certain particle sizes (500 μm and 900 μm) to the fibroin solution at a NaCl:fibroin wt/v ratio of 5:1. The mixture was placed in a 2.5 cm diameter mold and dried in a fume hood overnight. The dried NaCl-fibroin block was immersed in 70% alcohol for ∼30 minutes, followed by immersing in distilled water for three days to dissolve the salt, with changing the water every six hours. Before use, the silk fibroin scaffold was cut into 5 mm × 5 mm x 1 mm blocks, then sterilized by autoclaving for 15 minutes at 121 °C.

#### 2.3.2 Biomaterial from gelatin

The Gelatin hydrogel sponge from PI5 Gelatin (Nitta Gelatin, Osaka, Japan) was made based on the protocol described by Ishida et al. (2007) [3]. The gelatin was dissolved in MiliQ water to a final concentration of 3 wt% at 42 °C using a magnetic stirrer for ∼60 minutes, then a mixture of 25% glutaraldehyde (GA, Nakain inc.) in 3 wt% β-TCP (TAIHEI inc.) was prepared. The gelatin and GA - β-TCP was mixed using an AUTO CELL MASTER CM-200 at a speed of 500 rpm for 15 seconds. The foam mixture that was formed were placed in balance dishes (Bio-Bik inc.), then placed in a −80 °C freezer for 10 minutes, followed by incubation at 4 °C for 12 hours for the crosslinking process. The gelatin hydrogel sponge was shaken slowly in 0.1 M glycine at room temperature for one hour, followed by miliQ water twice for one hour each to remove unreacted GA and dried by freeze drying for three days. Before use, the gelatin hydrogel sponge was cut into blocks of 5 mm diameter and 1 mm height, then sterilized with ethylene oxide gas (EOG), followed by EOG-degassing gas at 40 °C overnight.

### 2.4 Scaffold morphology characterization

Scaffold pore morphology was observed using a Scanning Electron Microscope (SEM, SU3500 Hitachi High Technologies America, Inc). A sample of the scaffold was dried using a solution of Hexamethyldisilazane (HMDS Hitachi High Technologies America, Inc). The scaffold sample was first dried using Hexamethyldisilazane solution (HMDS, Electron Microscopy Science 16700), then it was coated with gold by sputter coating to give a conductive coating before observation with the SEM. The diameter of the pores were analyzed using imageJ software (developed by National Institute of Health, USA) by calculating at random 200 times on the field of vision for each sample.

### 2.5 Water content analysis of the scaffolds

Dried silk fibroin and gelatin hydrogel scaffolds were weighed and placed inside microtubes filled with distilled water. The wet scaffolds were then weighed after one and 24 hours using an analytical balance. The experiment was performed three times.

### 2.6 Biocompatibility test of the scaffolds

hMSC passage 5 were grown on sterile scaffold at 1 × 10^5^ cells/scaffold in 96 well plate. The cells were grown in standard medium and placed in an incubator at 37 °C, 5% CO_2_. On days 1, 3, 5, 7, and 14 of culture, the biocompatibilities of the scaffolds with the cells were evaluated using the MTT cytotoxicity assay. The standard medium was decanted and MTT reagent (Methylthiazolyldiphenyl-tetrazolium bromide, Sigma Aldrich M2128-1G) was added with a final concentration of 5 mg/mL, incubated at 37 °C for 4 hours in dark conditions. The MTT reagent was then removed and 100 μL/well Dimethyl Sulfoxide (DMSO, Sigma-Aldrich 276855) was added to dissolve the formazan crystal that was formed. The absorbance of the solution at 570 nm was read using a microplate reader (Bio-Rad).

### 2.7 Analysis of hMSC morphology on the scaffold biomaterial

hMSC passage 5 was grown on the scaffold biomaterials at 10^6^ cells/scaffold in 24 well plate. hMSC were grown in standard medium and placed in an incubator at 37 °C, 5% CO_2_ for three days. The medium of the culture was decanted and the sample was fixed with 500 μL 2.5% (v/v) glutaraldehyde in 0.1 M cacodylate buffer and incubated for two days at room temperature. Dehydration of the samples was performed by placing them in a series of ascending concentration of alcohol solution (30%, 40%, 50%, 60%, 70%, 80%, 90%, 100%), each for 15 minutes. The samples were dried using Hexamethyldisilazane solution (HMDS, Electron Miscroscopy Science 16700) and incubated overnight in a fume hood. The dried cell samples and scaffolds were coated with gold by sputter coating, then were observed using the Scanning Electron Microscope (SEM, SU3500 Hitachi High Technologies America, Inc).
